# Reparixin as a Potential Antiepileptogenic Agent: Modulation of the CXCL1–CXCR1/2 Axis and Seizure Activity in a Kindling Rat Model of Temporal Lobe Epilepsy

**DOI:** 10.3390/ijms26072831

**Published:** 2025-03-21

**Authors:** Nihan Çarçak, Nursima Mutlu, Elif Tuğçe Erdeve, Talat Taygun Turan, Özge Sarıyıldız, Canan Ulusoy, Elif Şanlı, Erdem Tüzün, Cem İsmail Küçükali, Laura Brandolini, Andrea Aramini, Marcello Allegretti, Filiz Onat, Lidia De Filippis

**Affiliations:** 1 Department of Pharmacology, Faculty of Pharmacy, Istanbul University, 34116 Istanbul, Türkiye; nihan.carcak@istanbul.edu.tr; 2 Department of Neuroscience, Institute of Health Sciences, Acibadem Mehmet Ali Aydinlar University, 34684 Istanbul, Türkiye; nursima.mutlu@acibadem.edu.tr (N.M.); taygun.turan@acibadem.edu.tr (T.T.T.); ozge.sariyildiz@acibadem.edu.tr (Ö.S.); 3 Department of Pharmacology, Institute of Health Sciences, Istanbul University, 34126 Istanbul, Türkiye; elif.tugce_erdeve@med.lu.se; 4 Department of Neuroscience, Aziz Sancar Institute of Experimental Medicine, Istanbul University, 34093 Istanbul, Türkiye; canan.ulusoy@istanbul.edu.tr (C.U.); elif.sanli@istanbul.edu.tr (E.Ş.); drerdem@yahoo.com (E.T.); cemsmile@istanbul.edu.tr (C.İ.K.); 5 R&D, Dompé Farmaceutici SpA, 67100 L’Aquila, Italy; laura.brandolini@dompe.com (L.B.); andrea.aramini@dompe.com (A.A.); marcello.allegretti@dompe.com (M.A.); 6 Department of Medical Pharmacology, School of Medicine, Acibadem Mehmet Ali Aydinlar University, 34684 Istanbul, Türkiye; 7 R&D, Dompé Farmaceutici SpA, 20122 Milano, Italy

**Keywords:** CXCL1, CXCR1/2 antagonist, amygdaloid kindling, reparixin, neuroinflammation, antiepileptogenic, antiseizure therapy

## Abstract

Chemokine (CXC motif) ligand 8 (CXCL8) is a pro-inflammatory chemokine binding to CXC motif receptors 1/2 (CXCR1/2). Patients with temporal lobe epilepsy (TLE) exhibit increased serum CXCL8 levels. CXC motif ligand 1 (CXCL1), a murine ortholog of CXCL8, has been implicated in seizure generation and neuronal loss. This study evaluated the antiepileptogenic and antiseizure effects of reparixin in amygdaloid kindling rat model of TLE. Reparixin was administered during the kindling period for 14 days, and seizures were induced twice daily via electrical stimulation. To assess the antiseizure effects, reparixin was administered to fully kindled animals, and stimulations were performed 24 and 48 h later. Levetiracetam, a broad-spectrum antiseizure drug, was administered intraperitoneally (i.p.) as positive control 1 h before each stimulation. Reparixin delayed secondary seizure generalization during kindling. Reparixin reduced seizure severity and after-discharge duration in fully kindled animals at 24 h from treatment initiation. CXCR1/2 and protein kinase B pathway proteins exhibited no significant changes; reparixin reduced the phospho-extracellular signal-regulated kinase (pERK)/ERK ratio in the cortex and hippocampus. CXCL1 expression was significantly decreased in the cortex. Reparixin exhibited antiepileptogenic and partial antiseizure effects by modulating the CXCL1–CXCR1/2 axis and reducing ERK signaling. Already in clinical trials on respiratory diseases, reparixin could be repurposed for epilepsy therapy.

## 1. Introduction

Epilepsy is a common neurological disorder affecting 1% of the total population. Epilepsy comprises recurrent, unprovoked seizures caused by abnormal neuronal firing, independent of a psychogenic event [1]. Most patients with epilepsy have seizures since childhood, indicating a higher sensitivity of the developing brain to seizures. Animal models of epilepsy have been extensively characterized to investigate the pathophysiology of epilepsy and to test novel antiepileptogenic and antiseizure drugs. The most common seizure models are generated by either chemical administration of convulsant drugs (e.g., pentylenetetrazol [PTZ] and kainic acid [KA]) or electrical stimulation (maximal electroshock [MES]) of target brain regions at their threshold values, with some differences in the form of ensuing seizures. Although PTZ induces absence seizures, MES induces tonic–clonic seizures. The recurrence of seizures and duration of clonus are the two main criteria for evaluating the efficacy of novel drugs.

The CXCL8 chemokine, also known as interleukin 8 (IL8), is notably altered in the sera or cerebral fluid of patients affected by seizures originating from autoimmune-associated epilepsy [2], epileptic encephalopathy with spike-wave activation in sleep [3], drug-resistant epilepsy [4], mesial temporal lobe epilepsy (MTLE) [5], and even COVID-19 infection [6], underlying a master role of IL8 in epileptogenesis. Thus, IL8 is a diagnostic biomarker [7] and clinical endpoint in antiseizure therapy.

**Figure 1:**
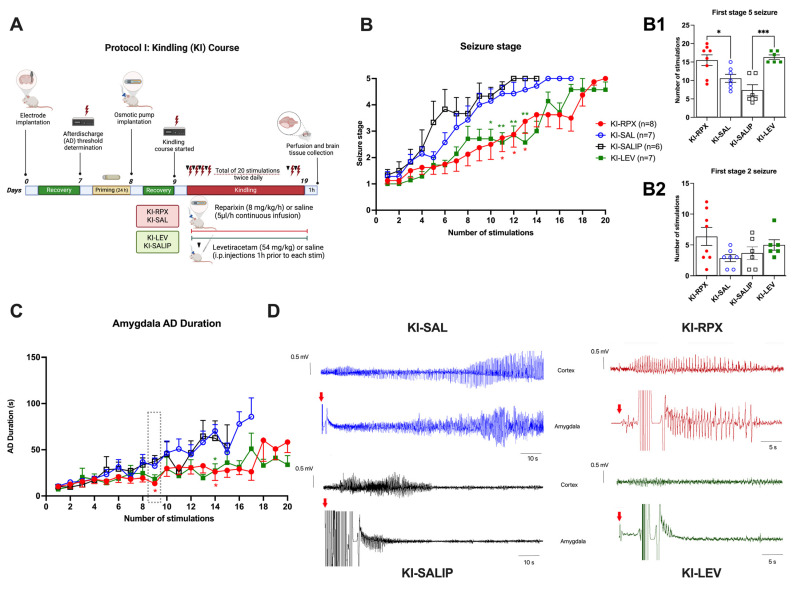
Antiepileptogenic effect of reparixin on kindling. Experimental plan (**A**) and seizure stages (**B**), the number of stimulations to reach first stage 5 seizure (**B1**) and stage 2 seizure (**B2**), and after-discharge (AD) durations (**C**) in the amygdala. The dotted line box indicates the 9th stimulation, during which EEG traces from the ipsilateral cortex and amygdala are represented for each group. In kindling (KI) groups treated with reparixin (KI-RPX, red traces) and levetiracetam (KI-LEV, green traces), after-discharges (ADs) induced by electrical stimulation of the amygdala are significantly shorter than in the control groups that received saline via an osmotic pump (KI-SAL, blue traces) or intraperitoneally (KI-SALIP, black traces). Red arrows indicate the timing of the electrical stimulation (**D**). Because of noise interference in EEG traces recorded from the amygdala, AD durations were evaluated using synchronized recordings from the ipsilateral cortex. Data are expressed as means ± standard errors of the mean. * *p* < 0.05, ** *p* < 0.01, *** *p* < 0.001. Experimental plan (**A**) was created in BioRender. https://BioRender.com where lightning bolts indicate electrical stimulations, and the black triangle represents the administration of levetiracetam one hour before each stimulation.

**Figure 2:**
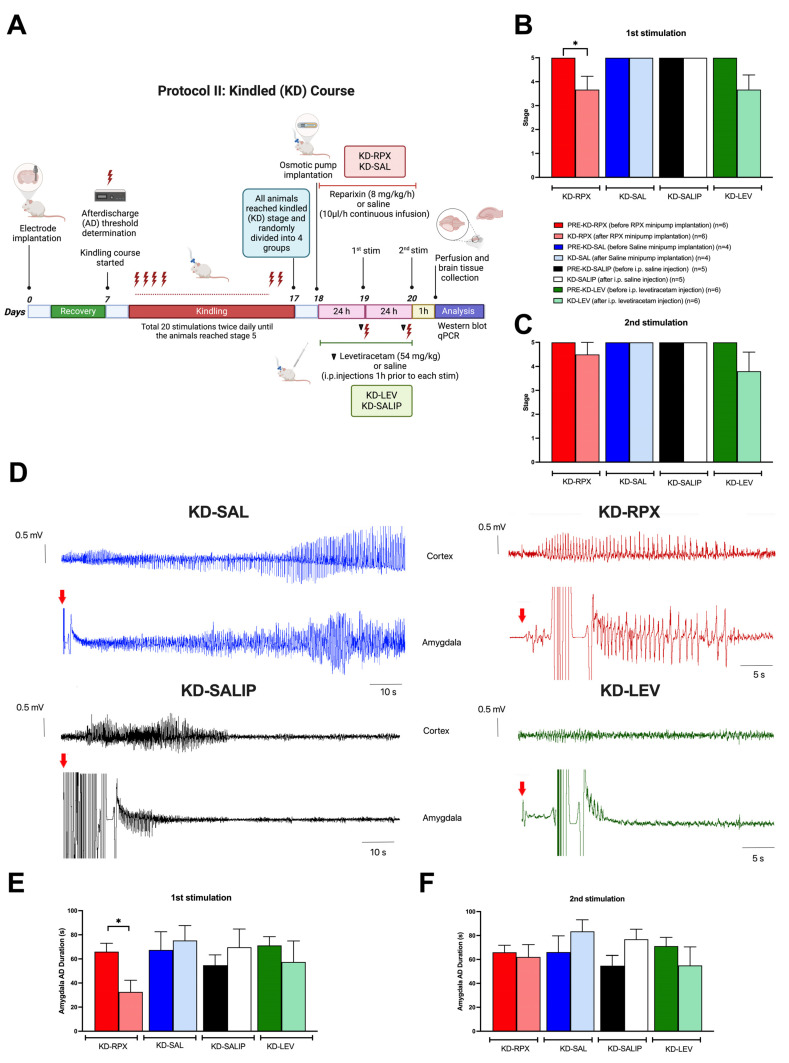
Antiseizure effect of reparixin in kindled (KD) rats. Experimental plan (**A**). The mean seizure stages after the first stimulation delivered 24 h after the osmotic pump implantation (**B**) and after the second stimulation delivered 48 h after the osmotic pump implantation (**C**). On the left, representative 110 s EEG traces from KD groups treated with saline via osmotic pump (KD-SAL, blue traces) or intraperitoneally (KD-SALIP, black traces) are indicated. On the right, representative 55 s traces from KD groups treated with reparixin (KD-RPX, red traces) or levetiracetam (54 mg/kg) (KD-LEV, green traces) are displayed. EEG traces, from both the amygdala and ipsilateral cortex showing ADs after the first stimulation (red arrows) delivered 24 h after the osmotic pump implantation (**D**). The mean AD durations in the ipsilateral amygdala after the first stimulation (**E**) and second stimulation (**F**). Data are expressed as means ± standard errors of the mean * *p* < 0.05. Experimental plan (**A**) was created in BioRender. https://BioRender.com, where lightning bolts indicate electrical stimulations, and the black triangle represents the administration of levetiracetam one hour before each stimulation.

CXCL1, a functional murine ortholog of CXCL8 (IL8), is an inflammatory cytokine involved in the enhancement of neurotransmitter release at the peripheral and central nervous systems, leading to hyperexcitability. A previous study [8] investigated the activation of the CXCL1–CXCR1/2 axis in epilepsy and its role in seizure generation using a murine model of acquired epilepsy induced by intra-amygdala kainate injection. In particular, mice were treated with continuous subcutaneous injection of an allosteric noncompetitive antagonist of CXCR1/2 (reparixin), and the number of recurrent seizures was remarkably reduced compared with that observed in untreated siblings [8]. Building on these findings, this study evaluated the antiepileptogenic (Protocol I) (Figure 1A) and antiseizure (Protocol II) (Figure 2A) effects of reparixin in a rodent model of chronic epilepsy. The kindling procedure, a well-established TLE model [9], was used to assess the development and progression of seizures.

## 2. Results

### 2.1. Antiepileptogenic Effect of Reparixin in the Kindling Process

Over the 20 kindling stimulations (Figure 1A), the KI-SAL (n = 7) and KI-SALIP (n = 6) animals, as control groups, exhibited a steady increase in seizure stages, whereas kindling-induced seizure stages in the KI-RPX (n = 8) and KI-LEV (n = 7) groups were significantly delayed (two-way analysis of variance [ANOVA], treatment effect F (3, 24) = 6.087; *p* = 0.0031) (Figure 1B). Continuous infusion of reparixin during the kindling course exerted a significant inhibitory effect on kindling acquisition, which induced a significantly lower seizure stage than that of the saline group at stimulation 11 (seizure stage 2.75 ± 0.5 vs. 4.6 ± 0.2 for KI-RPX vs. KI-SALIP; *p* = 0.03), stimulation 12 (2.87 ± 0.4 vs. 5.0 ± 0.0, respectively; *p* = 0.01), and stimulation 13 (3.37 ± 0.4 vs. 5.0 ± 0.0, respectively; *p* = 0.04) (Figure 1B). The administration of 54 mg/kg levetiracetam 1 h before each kindling stimulation also modified kindling progression compared with that observed in the saline group at stimulation 10 (seizure stage 2.71 ± 0.3 vs. 4.3 ± 0.3 for KI-LEV vs. KI-SALIP, respectively; *p* = 0.03), stimulation 11 (2.57 ± 0.3 vs. 4.66 ± 0.2, respectively; *p* = 0.003), stimulation 12 (2.85 ± 0.3 vs. 5.0 ± 0.0, respectively; *p* = 0.003), stimulation 13 (2.57 ± 0.3 vs. 5.0 ± 0.0; *p* = 0.002), and stimulation 14 (3.00 ± 0.3 vs. 5.0 ± 0.0, respectively; *p* = 0.007) (Figure 1A). The mean number of stimulations required to display first stage 5 seizures in the KI-SAL and KI-SALIP groups were not significantly different (Figure 1(B1)). However, in the KI-RPX and KI-LEV groups, the mean numbers of stimulations required to display first stage 5 seizures were 15.5 ± 1.5 and 16.3 ± 0.6, respectively, indicating a significant delay to secondary generalization of seizures during amygdala kindling compared with that observed in control animals (KI-SAL and KI-SALIP groups) (one-way ANOVA, treatment effect F (3, 23) = 10.68; *p* = 0.0001) (Figure 1(B1)). The mean number of stimulations to display first stage 2 seizures did not significantly differ between the treatment and control groups (Figure 1(B2)).

Two-way ANOVA treatment effect exhibited no significant differences among the groups in the after-discharge durations following the kindling stimulations (F (3, 24) = 0.8835, *p* > 0.05) (Figure 1C). However, Tukey’s multiple comparison test revealed that continuous reparixin infusions decreased the mean after-discharge durations from the amygdala at the 9th (13.75 ± 2.76 s vs. 37.55 ± 6.24 s for KI-RPX vs. KI-SALIP, respectively; *p* = 0.038) and 14th (26.11 ± 9.1 s vs. 70.367 ± 6.8 s for KI-RPX vs. KI-SAL, respectively; *p* = 0.026) stimulations. This difference was clearly visible in the representative electroencephalogram (EEG) traces during the ninth stimulation (Figure 1D). Tukey’s multiple comparison test also exhibited significant differences between the KI-LEV and KI-SAL groups at the 14th stimulation (27.38 ± 7.4 s vs. 70.367 ± 6.8 s for KI-LEV vs. KI-SAL, respectively; *p* = 0.018).

### 2.2. Antiseizure Effect of Reparixin in Kindled (KD) Rats

The KD rats (reached stage 5 seizures three times) were randomly divided into four groups: KD-RPX (n = 6), KD-SAL (n = 4), KD-LEV (n = 6), and KD-SALIP (n = 5). The KD-RPX and KD-SAL groups were implanted with an osmotic pump loaded with reparixin or vehicle (saline). KD animals were stimulated 24 h (first stimulation) and 48 h (2nd stimulation) after the implantation of osmotic pumps to observe the antiseizure effect of reparixin (Figure 2A). The KD-LEV and KD-SALIP groups were acutely administered levetiracetam (54 mg/kg, i.p.) and vehicle (saline, i.p.), respectively, 1 h before kindling stimulations and then stimulated 24 h (first stimulation) and 48 h (second stimulation) after the last kindling stimulation to test the anticonvulsant effect (Figure 2A). As shown in Figure 2B, reparixin significantly reduced the severity of seizures and after-discharge duration in fully KD animals. The mean seizure stages in the KD-RPX and KD-LEV groups were similar after the first stimulation. After the second stimulation delivered 48 h after osmotic pump implantation, seizure severity was not significantly reduced in either the reparixin- or levetiracetam-treated groups compared with the pre-stimulation level, which was stage 5 (Figure 2C). The mean seizure stage was 5.0 ± 0.0 for all vehicle-treated control groups, indicating that there was no effect on seizure severity (Figure 2B,C). The mean after-discharge duration from the amygdala was also significantly shorter in the KD-RPX group (32.6 ± 8.8 s, n = 6) than in the pre-stimulation levels (66.00 ± 6.3 s, n = 6) and in the vehicle group (75.3 ± 10.7 s; n = 4) (*p* < 0.05) 24 h after osmotic pump implantation (Figure 2D,E). No significant difference in the mean after-discharge duration 48 h was observed after osmotic pump implantation (Figure 2F).

### 2.3. Analysis of CXCL1–CXCR1/2 Signaling in a Rodent Model of Acquired Epilepsy

**Figure 3:**
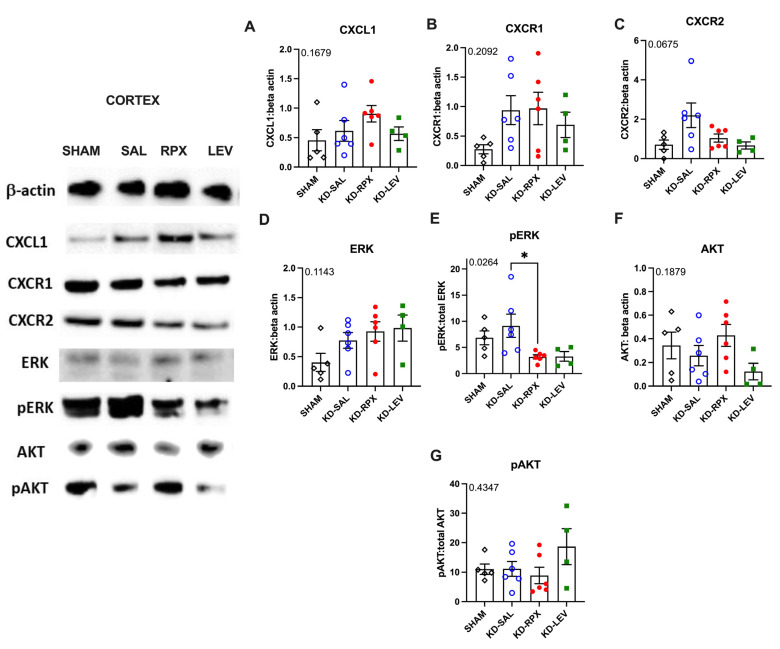
The effects of reparixin and levetiracetam on the protein expression levels of CXCL1 (**A**), CXCR1 (**B**), CXCR2 (**C**), ERK (**D**), p-ERK/ERK (**E**), and AKT (**F**), and the ratios of p-AKT/AKT (**G**) in the parietal cortex of the KD groups treated with saline (KD-SAL), reparixin (KD-RPX), or levetiracetam (KD-LEV) and sham-operated nonepileptic animals (SHAM). Representative immunoblotting bands of the parietal cortex showing increased pERK protein levels under epileptic conditions and reduced by reparixin treatment. * *p* < 0.05 by Tukey’s post-hoc test. The number on the upper left corner of each panel is the *p*-value obtained by ANOVA. Data are expressed as means ± standard errors of the mean.

**Figure 4:**
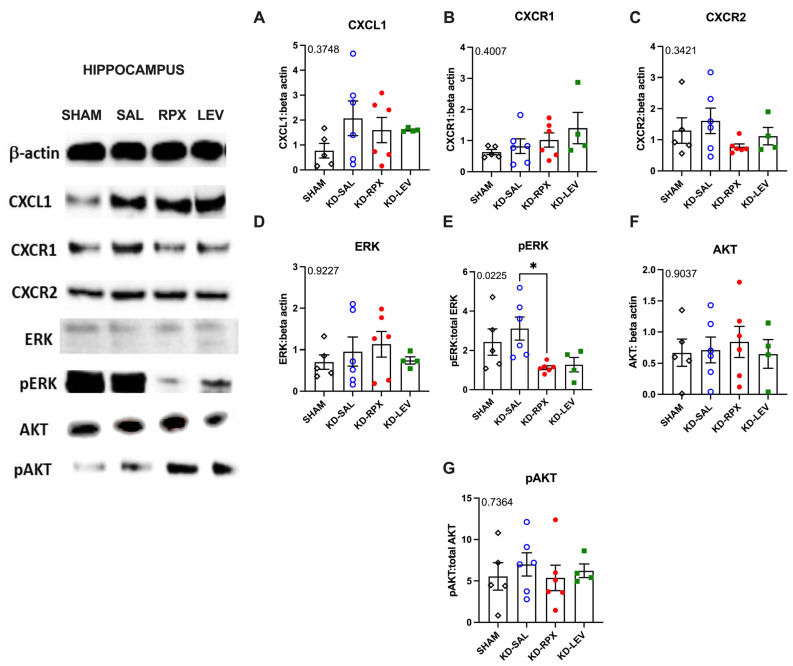
The effects of reparixin and levetiracetam on the protein expression levels of CXCL1 (**A**), CXCR1 (**B**), CXCR2 (**C**), ERK (**D**), p-ERK/ERK (**E**), and AKT (**F**) and the ratios of p-AKT/AKT (**G**) in the hippocampus of the KD groups treated with saline (KD-SAL), reparixin (KD-RPX), or levetiracetam (KD-LEV) and sham-operated nonepileptic animals (SHAM). Representative immunoblotting bands of the entire hippocampus showing increased pERK protein levels under epileptic conditions and reduced by reparixin treatment. * *p* < 0.05 by Tukey’s post-hoc test. The number on the upper left corner of each panel is the *p*-value obtained by analysis of variance (ANOVA). Data are expressed as means ± standard errors of the mean.

**Figure 5:**
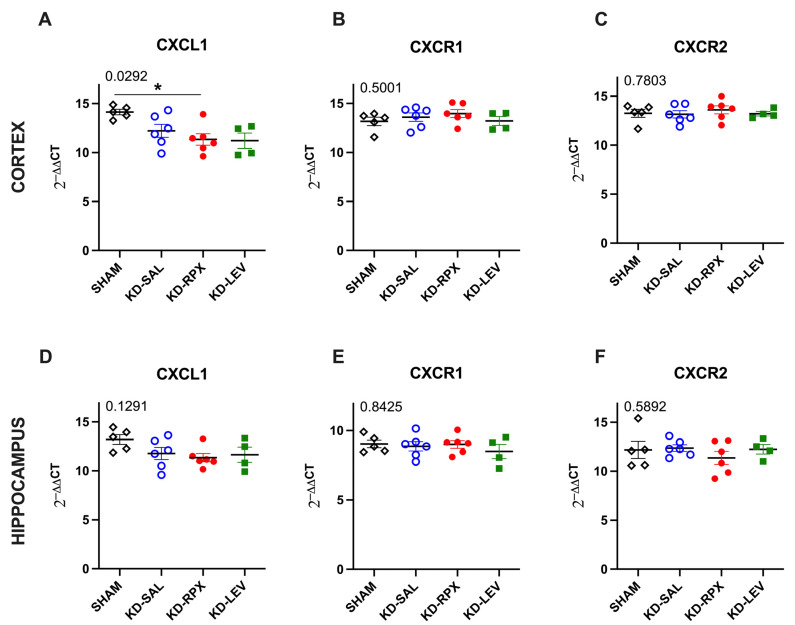
The effects of reparixin and levetiracetam on the mRNA expression levels of CXCL1, CXCR1, and CXCR2 in the cortex ((**A**), (**B**) and (**C**), respectively) and hippocampus ((**D**), (**E**) and (**F**), respectively) of the kindled (KD) groups treated with saline (KD-SAL), reparixin (KD-RPX), or levetiracetam (KD-LEV) and sham-operated nonepileptic animals (SHAM). * *p* < 0.05 by Tukey’s post-hoc test. The number on the upper left corner of each panel is the *p*-value obtained by analysis of variance (ANOVA). Horizontal lines indicate mean values. Data are expressed as means ± standard errors of the mean.

As described above, CXCR1/2 antagonism significantly reduced seizure severity and after-discharge duration in fully KD animals, showing a partial antiseizure effect. To determine the role of CXCL1–CXCR1/2 signaling in epileptogenicity, the activity of CXCL1–CXCR1/2 signaling was explored in KD animals, which may serve as a potential target for reparixin-mediated antiseizure therapy. To achieve this, we performed Western blotting and quantitative polymerase chain reaction (qPCR) to evaluate the protein and gene expression levels of CXCR1, CXCR2, and CXCL1 in two brain regions: the parietal cortex and total hippocampus. These analyses were performed on sham-operated, nonepileptic (SHAM), and KD groups. The KD groups were further treated with saline (KD-SAL), reparixin (KD-RPX), or levetiracetam (KD-LEV) as part of Protocol II. Furthermore, the protein and gene expression levels of intracellular effectors of CXCR1 and CXCR2 were examined, particularly focusing on the phosphorylated and non-phosphorylated forms of serine–threonine kinase (AKT), also known as protein kinase B, and extracellular signal-regulated kinase 1/2 (ERK1/2). Western blotting results revealed that the protein expression levels of CXCL1, CXCR1, CXCR2, AKT, and ERK and the phospho-AKT/AKT ratio exhibited no significant differences in either cortical (Figure 3A–G) or hippocampal tissues (Figure 4A–G). However, the phospho-ERK/ERK ratio was significantly downregulated in the cortex of reparixin-treated (KD-RPX) animals (*p* = 0.0264) and the hippocampus (*p* = 0.0225) (Figure 3E and Figure 4E). This indicates that although ERK-mediated pathway activation is significantly elevated under epileptic conditions as in the KD-SAL group, it is modulated by reparixin (Figure 3E and Figure 4E). These findings indicate that reparixin inhibits CXCR1/2-mediated signal transduction by reducing the phosphorylation of downstream ERK molecules. qPCR results revealed that cortical CXCL1 levels were significantly reduced in the reparixin-treated group (KD-RPX) compared with those in the other groups (Figure 5A). In the hippocampus, CXCL1 levels exhibited a similar decreasing trend relative to the sham group, although the difference was not statistically significant (Figure 5D). No significant differences in CXCR1 or CXCR2 expression levels were observed in either the hippocampus or cortex (Figure 5B,C,E,F).

## 3. Discussion

The role of CXCL1, a murine ortholog of the human chemokine CXCL8 (IL-8), and its receptors CXCR1 and CXCR2 in epilepsy was discussed in a previous study using a murine model [8]. Reparixin blocks CXCR1/2 receptors without affecting leukocyte migration induced by other chemoattractants [8]. It particularly inhibits neutrophil chemotaxis driven by CXCL1 and CXCL2 in rodents and neutrophil migration in humans [10].

In a previous study, we showed that in the KA model, reparixin administration for 2 weeks in mice with established chronic seizures reduced the average number of seizures by 2-fold compared with the pretreatment baseline [8]. The KA model seemed to perform equally well as the nonpharmacological MES model across drugs with different mechanisms of action [11], and both the KA, which is mostly associated with absence seizures, and MES, which is mostly associated with general tonic–clonic seizures, models can quantitatively predict human exposures resulting in epilepsy; thus, they are useful tools in early drug development.

In this study, the primary aim was to validate the antiepileptogenic effect of CXCR1/2 antagonism in TLE. Based on our results, we showed that reparixin is partially effective in a kindling model of TLE. Kindling development is an epileptogenesis process. This is the first study to demonstrate the antiepileptogenic effect of reparixin in an amygdala kindling rat model. The development of seizures induced by electrical stimulation was delayed by reparixin, although reparixin cannot completely stop the final kindling acquisition. Confirmed by previous results [12], a dose of 54 mg/kg of levetiracetam was also effective for the development of electrical kindling by reducing seizure severity and after-discharge duration induced by repeated amygdala stimulation. Reparixin significantly reduced the severity of seizures and after-discharge duration in fully KD animals, indicating that reparixin exerts a partial antiseizure effect.

To understand the role of CXCR1/2 activity in epileptogenicity, Western blotting and qPCR were performed to analyze the protein and gene expression levels of CXCL1, CXCR1, and CXCR2, intracellular effectors of CXCR1 and CXCR2, and phosphorylated and nonphosphorylated forms of AKT and ERK1/2 in the cortex and hippocampus.

Results revealed no difference in the protein levels of CXCL1, CXCR1, CXCR2, AKT, phospho-AKT, and ERK. The phospho-ERK/ERK ratios were significantly downregulated in both the hippocampus and cortex in the KD-RPX group compared with those observed in rats with seizures that did not receive treatment. Although increased protein levels of cortical CXCL1 and CXCR1 were observed in the KD-RPX group, these were not statistically significant. Furthermore, cortical CXCL1 mRNA levels were significantly decreased in the KD-RPX group. This may reflect a compensatory mechanism in response to increased protein expression.

Inhibition of the ERK pathway is associated with reduced seizures in several animal models of epilepsy [13,14,15]. Reparixin exerts anticancer effects, at least partially by inhibiting the phosphorylation of ERK molecules in cancer cells [16]. To the best of our knowledge, we have provided the first evidence that reparixin may reduce seizures through CXCR1/2-mediated signal transduction, as demonstrated by the decreased phosphorylation levels of ERK, the downstream protein of the CXCL1/CXCR1/2 signaling cascade.

Levetiracetam and reparixin induced similar changes in the CXCL1–CXCR1/2 pathway. However, these differences did not reach statistical significance for the levetiracetam group because of the small number of rats and insufficient statistical power. Although levetiracetam has been shown to alter phospho-ERK expression in a single study [17], the effects of this antiepileptic drug on the CXCL1–CXCR1/2 signaling pathway have not been reported. Thus, our results have provided preliminary evidence of a CXCL1-associated mechanism of action for this drug.

Reparixin may exert its antiepileptogenic and antiseizure effects by modulating neuronal excitability; therefore, discussing the mechanism by which CXCL1 activation of CXCR1/2 receptors influences neuronal excitability at multiple levels is crucial.

Because cerebral CXCL1 and NMDAR expression is correlated, we hypothesized that CXCL1 may modulate and enhance NMDAR activity through CXCR1/2 receptors and that inhibition of this interaction by reparixin may reduce NMDAR activity [18,19,20]. Moreover, α-amino-3-hydroxy-5-methyl-4-isoxazolepropionate receptor (AMPAR) and neuronal cholinergic receptor activity has been shown to be regulated by CXCR1/2 [21,22]. Furthermore, CXCL1 decreased astrocytic glutamate reuptake [23], increasing synaptic glutamate concentration, which in turn contributed to seizure activity. This suggests that CXCL1–CXCR1/2 signaling not only directly influences ionotropic glutamate receptors but also indirectly intensifies excitotoxicity by modulating astrocytic function. At the level of ion channels, CXCL1 has been shown to modulate neuronal excitability in dorsal root ganglion neurons by increasing sodium (Na⁺) currents, potassium (K⁺) currents, and transient receptor potential vanilloid-1 (TRPV1) channel function [24,25]. Furthermore, CXCR2 activation directly increases intracellular calcium (Ca^2^⁺) levels, which induce neurotransmitter release [26]. Reparixin, a CXCR1/2 antagonist, may counteract these effects, thereby reducing seizure susceptibility.

CXCL1 is a key chemotactic cytokine produced during inflammation and plays a crucial role in recruiting neutrophils to inflammatory sites [27]. In a murine model of viral encephalitis, astrocyte- and neuron-derived CXCL1, through its receptor CXCR2, facilitates neutrophil transmigration and blood–brain barrier (BBB) permeability [28]. Studies on animal models of peripheral inflammation, in which the brain is not initially affected, have demonstrated that infiltrating tumor necrosis factor-α (TNFα)-secreting neutrophils contribute to neuronal hyperexcitability [29]. Although the CXCL1–CXCR1/2 axis may indirectly modulate neuronal excitability through neutrophil infiltration and its effects on vascular tissue, its precise role in BBB disruption during epileptogenesis remains unclear. Understanding whether peripheral neutrophils contribute to seizure susceptibility and disease progression could provide further insights into the mechanisms by which CXCL1 signaling influences excitability and how reparixin may exert its antiepileptogenic effects.

Our findings suggest that reparixin influences the CXCL1/R1/R2 axis and should be further investigated for its possible therapeutic effects, likely mediated by immunomodulation of astrogliosis and microgliosis or secondarily by infiltrating immune circulating cells or to both [30,31].

## 4. Materials and Methods

### 4.1. Animals

This study was conducted on male 3–4-month-old *Wistar* rats (280–440 g). The rats were housed in Plexiglas cages in a temperature-controlled room (20 ± 3 °C) with a 12 h light–dark cycle. All animals were allowed free access to food and water. All procedures were approved by the Institutional Animal Care and Use Committee of Acıbadem University (ethical approval number: 2022/60), which conformed with the EU Directive 2010/63/EU for animal experiments. Every effort has been made to reduce the number of animals for experimentation and minimize pain and distress in animals.

### 4.2. Study Design and Experimental Groups

The study comprised two distinct experimental protocols, each aimed at investigating the specific therapeutic effects of reparixin. Protocol I was designed to evaluate the antiepileptogenic effects of reparixin, whereas Protocol II focused on assessing its antiseizure properties. In experimental Protocol I, we assessed the effects of reparixin on seizure development during the kindling process, and this group was referred to as the KI-RPX group (Figure 1A). Osmotic pumps were implanted 2 h before the initiation of kindling stimulation. Rats in the KI-RPX group (n = 8) received reparixin (8 mg/kg per h) via an osmotic pump for 14 days during the kindling procedure (KI-RPX, n = 8). The i.p. administration of levetiracetam, an antiseizure drug currently used to treat partial-onset, myoclonic, and generalized tonic–clonic seizures (54 mg/kg) [12,32], 1 h before each kindling stimulation was considered the reference standard (KI-LEV, n = 7). The negative control groups (KI-SALIP) received i.p. saline, as the vehicle of levetiracetam (n = 6). The KI-SAL group received the same volume of sterile physiological saline via an osmotic pump (n = 7). Kindling stimulations were conducted via bilateral electrodes implanted in the amygdala. Animals were stimulated daily, and seizure activity was recorded to assess the development of seizures over a 14-day period.

In experimental Protocol II, the antiseizure effects of reparixin were evaluated in KD (KD) animals that had experienced three consecutive stage 5 seizures (Figure 2A). Osmotic pumps (8 mg/kg per h) were then implanted into the KD animals following their third stage 5 seizure (KD-RPX, n = 6). To assess the antiseizure effects of reparixin, KD animals were subjected to electrical stimulation at 24 and 48 h after the implantation of osmotic pumps. The KD-LEV group served as a positive control, with levetiracetam i.p. administration 1 h before each stimulation (KD-LEV, n = 6). Regarding the negative control groups, the KD-SALIP group received saline (i.p.), as the vehicle of levetiracetam (n = 5); the KD-SAL group received the same volume of sterile physiological saline through an osmotic pump (n = 4). A group of animals, referred to as sham-operated (SHAM, n = 6), served as an additional negative control. These animals did not receive any treatment (neither drug nor saline) and were not subjected to electrical stimulation. This group was used as the control for brain tissue analysis.

### 4.3. Implantation of Electrodes and Determination of After-Discharge Threshold

*Wistar* rats were anesthetized with isoflurane (1–3% isoflurane in O_2_) and placed in a stereotaxic apparatus (Stoelting Model 51600, Wood Dale, IL, USA). The coordinates were obtained from the stereotaxic atlas [33], and the bregma was used as the reference point. An electrode (Plastics One, System MS 303, Protech International, Inc., Boerne, TX, USA) was implanted into the basolateral amygdala (2.6 mm posterior, 4.8 mm lateral, and 8.5 mm ventral from the bregma). Stainless steel screws were used to secure recording electrodes, which were placed bilaterally in the skull over the frontal cortex (2.0 mm anterior and 1.7 mm lateral from the bregma) and parietal cortex (6.0 mm posterior and 4.0 mm from the bregma). The electrodes were connected by insulated wires to a female microconnector for EEG recording. They were fixed to the skull using dental acrylic. All animals were allowed to recover from surgery for 7 days before the determination of the after-discharge threshold. After the 1-week recovery period, the animals were placed in Plexiglas cages. After stabilization, basal EEG was recorded for 20 min. To determine the after-discharge threshold, the animals were stimulated with an initial stimulus of 40 microA (biphasic square-wave pulses of 60 Hz, each 1 ms in duration, for a total duration of 1 s) and continued with 20-microA increments until after-discharges were induced on the EEG (Accupulser, A310, WPI, Saratosa, FL, USA). The after-discharge threshold is defined as a continuous train of ≥2 spikes occurring at least 1 s after the stimulation has ended. To summarize the determination of the after-discharge threshold, electrical stimulation is performed at an electrical current that causes an after-discharge on EEG. Therefore, before kindling or KD courses, the after-discharge threshold was determined in each rat by applying electrical stimulation to the basolateral amygdala. The animals in Protocol I were then randomly divided into five groups: KI-RPX (n = 8), KI-SAL (n = 7), KI-SALIP (n = 6), and KI-LEV (n = 7). The KI-RPX and KI-SAL groups were implanted with osmotic pumps (Alzet model 2ML2, velocity 5.0 μL/h for 14 days) loaded with either reparixin (8 mg/kg/h) or sterile saline (0.9% NaCl) before the kindling process. The kindling procedure was started 24 h after osmotic pump implantation (Figure 1A). Furthermore, the KI-LEV and KI-SALIP groups received i.p. injections of levetiracetam (54 mg/kg) or saline during the kindling process; the KI-LEV group was considered the positive control group. The kindling development rate was compared between the groups.

In Protocol II, KD rats that had reached stage 5 seizures three times were randomly divided into four groups: KD-RPX (n = 6), KI-SAL (n = 4), KI-SALIP (n = 5), and KI-LEV (n = 6). The KD-RPX and KD-SAL groups were implanted with osmotic pumps (Alzet model 2ML1, velocity 10 μL/h for 7 days) loaded with 2.2 mL reparixin (8 mg/kg/h) solution or sterile saline (0.9% NaCl). The KD-LEV and KD-SALIP groups received i.p. injections of levetiracetam (54 mg/kg) and vehicle (saline), respectively, 1 h before electrical stimulation. All animals then received additional electrical stimulation at 24 h (first stimulation) and 48 h (second stimulation) after reaching a fully KD state (Figure 2A).

### 4.4. Kindling Protocol

The rats received electrical stimulation at their after-discharge threshold current intensity twice daily, with an inter-stimulation interval of at least 4 h, 5 days per week until a total of 20 stimulations (i.e., 14 days). Behavioral manifestations of the seizures induced by the electrical stimulations were classified according to the Racine scale [34]: stage 1, facial clonus; stage 2, head nodding; stage 3, contralateral forelimb clonus; stage 4, bilateral forelimb clonus and rearing; and stage 5, rearing and falling. The rats were KD to full stage 5 seizures. The criterion of KD was three consecutive stage 5 seizures. EEG was amplified through a BioAmp ML 136 amplifier (PowerLab8S ADI Instruments, Oxfordshire, UK), with bandpass filter settings of 1–40 Hz, and recorded and analyzed using Chart v.8.1 (PowerLab8S ADI Instruments, Oxfordshire, UK). The after-discharge durations from the amygdala and cortex were assessed from the EEG recordings offline.

### 4.5. Drug Preparation

Reparixin (provided by Dompé Farmaceutici SpA, L’Aquila, Italy) was dissolved in sterile physiological saline under sterile conditions. To achieve continuous and controlled dosing, reparixin was administered using an osmotic pump for the KI-RPX and KD-RPX groups (Alzet model 2ML2: 5.0 μL/h, 14 days and 2ML1: 10 μL/h, respectively) for 7 days. The osmotic pump (Alzet model 2ML2 or 2ML1, Cupertino, CA, USA) was filled with reparixin solution (8 mg/kg/h; dissolved in 2.2 mL sterile saline) or its vehicle (sterile saline) using a sterile syringe. The concentration of the reparixin solution was calculated to deliver 8 mg/kg/h, a dose proved to be effective in previous preclinical studies [8,35,36]. Reparixin was always freshly prepared before each osmotic pump preparation. Then, the osmotic pumps were kept in an incubator at 37 °C for 24 h before implantation, following the manufacturer’s instructions. Levetiracetam was supplied by Abdi İbrahim Pharmaceuticals, Istanbul, Türkiye (Epixx 5 mg/5 mL) and injected at a dose of 54 mg/kg (i.p.). It exerted potent anticonvulsant and antiepileptogenic activities against both focal and secondarily generalized seizures [12,37].

### 4.6. Osmotic Pump Implantation

The osmotic pumps were surgically implanted subcutaneously on the back under general anesthesia (1–3% isoflurane in O_2_). A small incision was made between the scapulae, and a subcutaneous pocket was created by gently separating the connective tissue under sterile conditions. The pump was then inserted into the pocket, and the skin was carefully sutured to minimize discomfort to the animal. Upon awakening from anesthesia, the rats were returned to their home cages. The entire procedure was completed in 10 min. Postoperative care included wound monitoring, weight tracking, and intramuscular administration of saline and paracetamol.

### 4.7. Brain Tissue Preparation

At the end of the protocol, 1 h after the final kindling stimulation, the rats were deeply anesthetized with ketamine (100 mg/kg, i.p.) and xylazine (10 mg/kg, i.p.). They were then perfused with 50 mM ice-cold phosphate-buffered saline (PBS) (pH 7.4). Subsequently, the brains were removed from the skull, and the two hemispheres were separated. The right hemisphere, ipsilateral to the stimulating electrode, was kept for histological verification, immediately frozen in liquid nitrogen, and stored at −80 °C until assay. The entire hippocampus and parietal cortex from the contralateral hemisphere (left) were dissected out on ice (4 °C), immediately frozen in liquid nitrogen, and stored at −80 °C until molecular assays.

### 4.8. Western Blotting

The cortex and hippocampus tissues from the left hemisphere were mixed with zirconium oxide (Next Advance, Troy, NY, USA, 1.5 mm, #ZrOB15,) at a 1:1 ratio in 1.5 mL tubes. In each tube, a mixture of 500 μL T-PER tissue extraction reagent (Thermo-Fisher Scientific, Waltham, MA, USA, #78510) and 5 μL Halt protease inhibitor cocktail (100×, Thermo-Fisher Scientific, Waltham, MA, USA, #78429) was added. The tissues were homogenized for 3 min. The samples were then centrifuged with 10,000× *g* in 5 min at +4 °C to separate the beads and homogenates. The homogenates were collected in new tubes, and the protein concentration was measured using Qubit (Invitrogen, Waltham, MA, USA) (Supplemental Table S1). The proteins were diluted with dH_2_O and aliquoted to 15 μg in each sodium dodecyl sulfate-polyacrylamide gel electrophoresis well in 26 μL. This volume was chosen according to the mixture volume LDS sample buffer (4×) (10 μL) (Invitrogen, MA, USA, #NP0007) and NuPAGE reducing agent (10×) (4 μL) (Invitrogen, MA, USA, #NP0004) so that the final loading volume for each well was 40 μL/well. After adding LDS sample buffer and NuPAGE reducing agent, the protein was aligned at 70° for 10 min. Then, the samples were loaded onto Bis-Tris gel 4–12% (Invitrogen, MA, USA, #NW04122BOX) and run at 220 V for 20 min. After that, the samples were transferred to the membrane using an iBlot 2 Nitrocellulose membrane (Invitrogen, Waltham, MA, USA, #IB23001). Antibodies were applied using the iBind Flex Western Blot system (Thermo-Fisher Scientific, Waltham, MA, USA, #SLF200) or overnight incubation at 4 °C for primary antibodies following a 2 h incubation at room temperature for secondary antibodies (Supplementary Table S2). The membranes were washed with PBS (3 × 10 min), and Western blot bands were visualized using a chemiluminescent substrate kit (SuperSignal™ West Pico PLUS Chemiluminescent Substrate, Thermo-Fisher Scientific, Waltham, MA, USA, #34580) after incubation. Densitometric analysis of Western blot bands was performed using computer software (iBrightAnalysis Software, Thermo-Fisher Scientific, version 5.2.0.0, Waltham, MA, USA) normalized to beta actin.

### 4.9. Real-Time PCR

Total RNAs were isolated from the brain homogenates using a PureLink RNA Mini Kit (Invitrogen, Waltham, MA, USA, #1218301BA). The concentration and purity of the isolated RNAs were measured, and samples with a 260/280 nm ratio of ≥1.9 were selected for the experiments. RNA samples were stored in a deep freezer at −80 °C. A High-Capacity cDNA Reverse Transcription Kit (Applied Biosystems, Waltham, MA, USA, #4368814) was used for cDNA synthesis from total RNA samples. Real-time PCR was performed for the evaluation of gene expression using the QuantGene 9600 Real-Time Thermalcycler PCR device (Bioer Technology, Hangzhou, China) with the 2× AMPIGENE qPCR Probe Mix Hi-ROX (Enzo Life Sciences, Farmingdale, NY, USA, #ENZ-NUC106), according to the manufacturer’s instructions. The temperature conditions for PCR were as follows: initial PCR activation phase at 95 °C for 10 min, denaturation phase at 95 °C for 15 s, annealing phase at 62 °C for 15 s, and extension phase at 72 °C for 20 s. Relative quantification was evaluated with reference to glyceraldehyde 3-phosphate dehydrogenase (GAPDH) and calculated using the cycle threshold (CT) method [2 − (Δ*CT* gene of interest − Δ*CT GAPDH*)]. Table 1 presents the primer sequences.

### 4.10. Data Analysis

Statistical analyses were performed using GraphPad Prism version 9.3 (GraphPad Software, MA, USA). All quantitative data in the text and figures are expressed as mean ± standard errors of the mean. Two-way ANOVA, followed by Tukey’s multiple comparisons test, was used to analyze drug × time interaction on seizure stage and after-discharge duration. The unpaired Student’s *t*-test was used to analyze the seizure stages and after-discharge durations in the different KD groups. One-way ANOVA, followed by Tukey’s multiple comparison test, was used to analyze the number of stimulations required to reach first stage 5 seizures and the Western blotting and real-time qPCR results. *p*-values < 0.05 were used to denote statistical significance.

## 5. Conclusions

This study supports the idea that reparixin treatment can modify seizure development in amygdala kindling rat models, showing antiepileptogenic and antiseizure effects. There are no data on the antiepileptogenic effect of reparixin in humans yet. Thus, future studies should explore whether the administration of reparixin could extend the therapeutic efficacy clinically.

Taken together, these findings suggest that investigating the mechanism underlying the antiepileptogenic effect of reparixin can be targeted with additional further studies (particularly in the kindling experiment).

## Figures and Tables

**Table 1:** Primers used in gene expression analysis by real-time PCR.

Gene	Frw Primer (5′-3′)	Rev Primer (5′-3′)
*CXCL1*	*CTCGCTTCTCTGTGCAGC*	*AGTGTGGCTATGACTTCGGT*
*CXCR1*	*ACCGATGTCTACGTGCTGAA*	*GATGGCCAGGTATCGATCCA*
*CXCR2*	*TGTCCCTGCCCATCTTCATT*	*CGAGGACCACAGCAAAGATG*
*GAPDH*	*ATGGTGAAGGTCGGTGTGAAC*	*TGTAGTTGAGGTCAATGAAGG*

## Data Availability

The data will be provided by the request from other researchers.

## Supplementary Materials

The following supporting information can be downloaded at: https://www.mdpi.com/article/10.3390/ijms26072831/s1.
