# Supplementary material for: Reparixin as a Potential Antiepileptogenic Agent: Modulation of the CXCL1–CXCR1/2 Axis and Seizure Activity in a Kindling Rat Model of Temporal Lobe Epilepsy
Source: Int J Mol Sci. 2025 Mar 21;26(7):2831. doi: 10.3390/ijms26072831 (PMC11989020; doi:10.3390/ijms26072831)
Supplement: Supplementary file 1 [file ijms-26-02831-s001.zip › ijms-3489684-supplementary.pdf]

SUPPLEMENTARY INFORMATION

REPARIXIN AS A POTENTIAL ANTI-EPILEPTOGENIC AGENT: MODULATION OF CXCL1-CXCR1/2 AXIS AND SEIZURE ACTIVITY IN KINDLING MODEL OF TEMPORAL LOBE EPILEPSY

Nihan Carcak[1,2], Nursima Mutlu[2], Elif Tuğçe Erdeve[3], Taygun Turan[2], Özge Sarıyıldız[2], Canan Aysel Ulusoy[4], Elif Sanli[4], Erdem Tuzun[4], Cem Ismail Kucukali[4], L. Brandolini[5], A. Aramini[5], M. Allegretti[5], F. Onat[2,6*], L. De Filippis[5,7*]

1. Department of Pharmacology, Faculty of Pharmacy, Istanbul University, Istanbul, Türkiye
2. Institute of Health Sciences, Department of Neuroscience, Acibadem Mehmet Ali Aydinlar University, Istanbul, Türkiye.
3. Institute of Health Sciences, Department of Pharmacology, Istanbul University, Istanbul, Türkiye
4. Department of Neuroscience, Aziz Sancar Institute of Experimental Medicine, Istanbul University, Istanbul, Türkiye.
5. R&D, Dompé Farmaceutici SpA, L'Aquila, Italy
6. Department of Medical Pharmacology, School of Medicine, Acibadem Mehmet Ali Aydinlar University, Istanbul, Türkiye.
7. R&D, Dompé Farmaceutici SpA, Milano, Italy

Supplementary table S1. Protein concentrations (ng/ul)

Samples	Cortex	Hippocampus
**Sham 2**	1.65	1.70
**Sham 3**	1.50	1.79
**Sham 4**	1.39	2
**Sham 5**	1.5	1.73
**Sham 10**	1.61	2.39
**KD-SAL 2**	1.75	2
**KD-SAL 3**	1.53	1.7
**KD-SAL 4**	1.56	1.65
**KD-SAL 6**	1.4	1.35
**KD-SAL 4**	1.57	1.66

**KD-RPX 3**	1.58	1.98
**KD-RPX 5**	1.55	2.6
**KD-RPX 6**	1.26	1.33
**KD-RPX 8**	1.39	1.34
**KD-RPX 9**	1.29	1.35
**KD-LEV 3**	1.39	1.6
**KD-LEV 4**	1.61	1.6
**KD-LEV 5**	1.46	1.79
**KD-LEV 6**	1.26	1.45

Supplementary Table S2. Antibodies used in western blot studies. Dilutions were prepared in 2.5% BSA-TBS- 0.05 Tween 20.

Antibodies	Brand	Catalog Number	Dilution ratio
**Rabbit anti-rat CXCR1**	Invitrogen	PA5-95749	1:2000
**Rabbit anti-rat CXCR2**	Invitrogen	PA5-102662	1:1000
**Rabbit anti-rat CXCL1**	Invitrogen	PA5-86508	1:1000
**Rabbit anti-rat phospho-AKT**	Invitrogen	PA5-85513	1:1000
**Rabbit anti-rat AKT**	Abcam	ab25893	1:1000
**Rabbit anti-rat phospho-ERK**	Invitrogen	44-680G	1:2000
**Rabbit anti-rat ERK**	Invitrogen	13-6200	1:1000
**Rabbit anti-rat Beta actin**	Invitrogen	PA1-183	1:2000
**Goat anti-rabbit IgG,HRP**	Invitrogen	32460	1:3000
